# Glaucoma Treated with Eserine

**Published:** 1885-12

**Authors:** S. O. Richey

**Affiliations:** Washington, D. C.


### A CASE OF CHRONIC SIMPLE GLAUCOMA TREATED WITH ESERINE. S. O. RICHEY, M. D., *Washington, D. C.*

[Read before the Medical Society of the District of Columbia.]

This is not written with a hope of adding anything new to the subject, nor with an intention to fully discuss the varying views of its pathology, but only to report a case, in my judgment, worthy of record, because it is one of "the type of the whole group of glaucomatous diseases."

On January 21, 1885, I examined Mrs. F., aged 37 years, whose left eye was blind from absolute glaucoma; the vision of her right eye was $\frac{20}{200}$, and much blurred. She knows the left eye to have been blind for seven years; the right has been affected for that length of time, but she does not know whether it has been diseased for a longer time than seven years or not. When she was about eighteen years old she noticed the halo, and had flashes of light, but these do not now continue. The right eye has been sensitive to light for seven years, but she has never had any pain in either of her eyes.

Just before Christmas she sewed quite a good deal, using her mother's glasses, before which time she had not used her eyes for a year at close work. "The room seems full of smoke," as she looks about it now. She was frightened into consulting a physician by finding that, although she could see the form, she could not distinguish the face of her pastor in the pulpit a few days since.

A lens serves only to interrupt the vision of the right eye. The discs are well cupped with sharp outlines. The excavation is deeper in the left eye, and the nerve is atrophied. Tension + 3; both feel like small marbles; the media are clear, the pupils half-dilated and regular; the anterior chambers are shallow, and the corneæ anæsthetic. The field of vision is limited to the nasal half.

Her general health is bad; her digestion is poor, associated with great mental depression. The idea of an operation creates dread, and the treatment adopted is sulphate of eserine (gr. ¼ to f ℥i), applied twice a day, with dry cups to the temples.

January 25th. Vision of right eye, $\frac{20}{70}$; tension + 2. There is no very marked change in the size of the pupils, nor in the depth of the anterior chambers. Liquor potassæ arsenitis in gtt. ii doses was given *ter in die*, to correct fermentation, and kal. iod. after meals, to hasten tissue change, are added.

January 29th, the pupils are normal in size, but still sluggish, due now probably to the eserine. Vision, $\frac{20}{40}$. The improvement constant and gradual until March 28th, when vision $\frac{20}{20}$; the tension normal. The field is not altered.

November 11th, 1885, eleven months after the first interview, the field is not changed; the tension is normal; vision, in the right eye, is slightly less than normal, and her general condition is much improved. She has been advised to persist, for a time, in the occasional use of the myotic, because the condition is a very chronic one; that she must avoid mental excitement, and preserve her general bodily health in the hope that this interruption to the progress of the disease may be permanent. Iodide of potash was exhibited with a view to breaking up any adhesions of the iris that might exist, upon the basis of its action in the treatment of posterior synechia. This also has been continued at intervals.

The special features of the case are: the age of the patient when first affected (eighteen years); the great amount of tension tolerated for so long a time, and the degree of vision recovered without operation. There was no hypermetropia, and the necessity for convex lenses at her age may be explained by the probability of the suspensory ligament being put so much on a stretch by pressure that accommodation was interfered with, or that the retina, being obtunded by pressure, required larger images than usual.

Mr. Henry Powers * says: "Eserine is a valuable remedy, and he is inclined to doubt whether some of the results attributed to sclerotomy might not really have been due to the use of eserine. If the pupil responded to eserine, he would certainly continue its use and not operate at that time."

* O. Review, 1882, p. 262.

This affection, presenting itself in so many ways, is least hopeful in the chronic simple form, which rarely announces its presence before the age of fifty years, and makes slow, insidious progress, generally without any inflammation, as in this case, and commonly proves fatal to vision, whatever the treatment. Though many expedients are helpful, it is very intractable.

The methods of relief are surgical, except the use of myotics; iridectomy, sclerotomy, and stretching the supra-trochlear nerve,—the last suggested by Badal, of Bordeaux, in 1882,—each founded upon a rational theory of the pathogeny of the affection.

Increase of tension is pathognomonic of the disease, and whether this is due to hypersecretion or an impeded outflow of the intra-ocular fluids, opinions differ. Priestly Smith* says: "It is certain that glaucoma is essentially due to pressure; that it is cured by reduction of tension, and that this reduction is due to restored filtration."

It was upon this theory of the nature of glaucoma that Græfe performed iridectomy, to relieve swelling of the ciliary folds, and the adhesions of the iris at the iritic angle, which obstruct the entrance of the intra-ocular fluids to the canal of Schlemm, though Wolfe† claims the benefit from iridectomy to be due to the removal of a section of the diseased nerves, upon the theory of nutritive changes in the nerve-centers.

The cupping of the optic disc is held by Mooren‡ to be due to changes external to the eye, and he is convinced that "The presence or absence, and breadth and depth of the excavation are not dependent in any way on the degree of increase of in-

---

* Ophthalmic Review, 1882, p. 261.

† Wolfe, On Diseases and Injuries to the Eye.

‡ Archiv. Ophth., March, 1884.

tra-ocular pressure ; . . . . that even the most brilliant therapeutic results will not alter the fact that in glaucomatous excavation of the optic nerve, or in the excavation of glaucoma simplex, with or without inflammatory symptoms, we have to deal with some anomaly of nutrition of the optic nerve."

All observers agree that there is no fixed relation between intra-ocular tension and the disc-cupping, though when there has been great tension with little or no cupping, the fact has been attributed to the greater power of resistance of the optic papilla.

Gowers* records the fact that " Glaucoma is sometimes observed in cases in which there was long standing liability to unilateral neuralgia, . . . that optic atrophy has resulted from the same cause, and that irritation of the fifth nerve may increase intra-ocular tension." Douders† refers to the fifth nerve as controlling intra-ocular secretions, and Grünhagen and Hippel‡ found that irritation of the trigeminus produced increased intra-ocular pressure.

From the foregoing it may be seen that there are at least two methods of accounting for glaucoma : the pressure theory and that of a neurosis. These are not necessarily antagonistic, for each may explain one stage of the same attack. Each attack must have an initial stage, as, in secondary glaucoma, a dislocated lens, or an intra-ocular hemorrhage; so, in primary simple glaucoma, some disturbance of the sympathetic system may interfere with the balance existing between the secretion and filtration of intra-ocular fluids, and by increasing tension, inaugurate a " vicious circle."

Iridectomy is the most reliable of all the measures adopted

---

* Medical Ophthalmoscopy, p, 172.

† Klin. Monatsblatt fur Augenheilkunde, 1884, p. 434.

‡ Arch. fur Ophthal. B. xiv. 3, p. 219.

in glaucoma, though it sometimes fails, and in some cases the probability of hemorrhage forbids it. Its influence may be explained in accordance with either theory, for it acts in both ways, relieving tension, and by removing a section of the nerve, but Mooren thinks it "useless in all cases of glaucomatous disease which do not originate in the eye but in the centre of nutrition of the optic nerve." Badal's operation of stretching the supra-trochlear nerve is based upon the theory of a neurosis, and has been efficient in some cases—even in some which have defied other efforts at relief. It is, however, not reliable, and should be only a last resort.

Sclerotomy reduces tension by opening a way between the aqueous chamber and the sub-conjunctival space, producing an anterior staphyloma and establishing permanent drainage. It is likely to be followed by distortion of images in consequence of the staphyloma. The necessity of using a myotic diminishes the importance of the procedure, and the objection has been made that it excites sympathetic inflammation. As the incision must be made within the sclerotic, it is probable the ciliary region has been wounded in such instances.

Priestly Smith claims the essential part of this operation and iridectomy to be the same, only the incision is more favorably made in sclerotomy; that when the incision is made as peripheral in iridectomy, it is the more reliable operation, and the effect is more permanent, although it is not so safe in cases where there is immediate danger of hemorrhage. Argyle Robertson's operation of trephining the cornea has been successful in a few cases, but has not at any time met with general adoption. Paracentesis is a measure to gain time, or to discover the amount of vision that may be recovered by an iridectomy in acute glaucoma.

Eserine, it is said, gives its best results in acute primary glaucoma, and may entirely relieve the eye, but while it sometimes improves the condition in simple chronic glaucoma, this improvement will probably not be permanent.

Williams,* of Boston, has not an impression favorable to the use of eserine in glaucoma, and doubts its efficiency in this affection in the hands of others. This view is one for which it is difficult to account, as the drug has unquestionable powers to reduce tension.

Snell† believes that eserine not only improves vision but arrests the progress of chronic simple glaucoma.

I have not found it produce follicular conjunctivitis, to which Wecker refers, because, I think, I have not been accustomed to use it in so strong solution. The milder solutions have in my hands been as useful as the strong ones and without any misadventure, though they must be used more frequently. The frequency of application of a mild preparation lessens the tendency to spasmodic myosis, The action is more gentle and less likely to produce congestion of either the conjunctiva or the iris. Eserine is preferable to pilocarpine, because this disposition to spasm may be controlled, and it maintains myosis for a longer period.

---

### BLINDNESS FOLLOWING HEMORRHAGE.

*By* BOERNE BETTMAN, M. D.

(Read before the Chicago Society of Ophthalmology and Otology.)

Cases of amaurosis following hemorrhage, although not infrequent, are, owing to the darkness enshrouding their pathology, full of interest. The two cases which I will report to the Society this evening offer certain symptoms which point

---

* Williams, Diagnosis and Treatment of Diseases of the Eye. 1881.

† On Eserine and Pilocarpine in Glaucoma, and Eserine in Ocular Neuralgia.

